# Search for Compounds with Hypoglycemic Activity in the Series of 1-[2-(1*H*-Tetrazol-5-yl)-R^1^-phenyl]-3-R^2^-phenyl(ethyl)ureas and R^1^-Tetrazolo[1,5-*c*]quinazolin-5(6*H*)-ones

**DOI:** 10.3797/scipharm.1507-14

**Published:** 2015-10-10

**Authors:** Oleksii M. Antypenko, Sergiy I. Kovalenko, Galina O. Zhernova

**Affiliations:** 1 Department of Organic and Bioorganic Chemistry, Zaporizhzhya State Medical University, Mayakovsky 26 ave., 69035, Zaporizhzhya, Ukraine; 2 Department of Pharmacognosy, Pharmacology and Botany, Zaporizhzhya State Medical University, Mayakovsky 26 ave., 69035, Zaporizhzhya, Ukraine

**Keywords:** 1-[2-(1*H*-Tetrazol-5-yl)-R^1^-phenyl]-3-R^2^-phenyl(ethyl)ureas, Cyclization, Hypoglycemic activity, Molecular docking, Synthesis

## Abstract

Methods of 1-[2-(1*H*-tetrazol-5-yl)-R^1^-phenyl]-3-R^2^-phenyl(ethyl)ureas and R^1^-tetrazolo[1,5-*c*]quinazolin-5(6*H*)-ones synthesis were designed. IR, LC-MS, ^1^H NMR, and elemental analysis data evaluated the structure and purity of the obtained compounds. Different products, depending on the reaction conditions, were distinguished and discussed. The preliminary hypoglycemic activity of 36 synthesized compounds was revealed. Docking studies to 11β-hydroxysteroid dehydrogenase 1, γ-peroxisome proliferator-activated receptor, and dipeptidyl peptidase-4 were conducted. Eight of these substances were further tested on glucocorticoid-induced insulin resistance models, namely glucose tolerance, oral rapid insulin, and adrenalin tests. One of the most active compounds turned out to be tetrazolo[1,5-*c*]quinazolin-5(6*H*)-one 3.1, exceeding the reference drugs Metformin (50 and 200 mg/kg) and Gliclazide (50 mg/kg).

## Introduction

Oral drugs used to treat type 2 diabetes mellitus (T2DM) are divided into six big groups: biguanides (e.g. Metformin), sulfonylureas (e.g. Glimepiride), meglitinides (e.g. Repaglinide), thiazolidinediones (e.g. Pioglitazone), dipeptidyl peptidase IV inhibitors (DPP4, e.g. Linagliptin), and α-glucosidase inhibitors (e.g. Acarbose). Each group has disadvantages with side effects. In particular, the inability of complete glycohemoglobin (HbA1c) level control. Currently, the most commonly used drugs with the exception of insulin can reduce the HbA1c level by just 1%. It is not enough for patients who have long-term diabetes and whose level of HbA1c significantly exceeds the normal rate. Therefore, a solution to this problem can be found in the creation of combinatorial drugs or drugs with an entirely new mechanism of action. These drugs must be able to provide prolonged hypoglycemic effects and influence not only the symptoms, but also their causes.

**Fig. 1:**
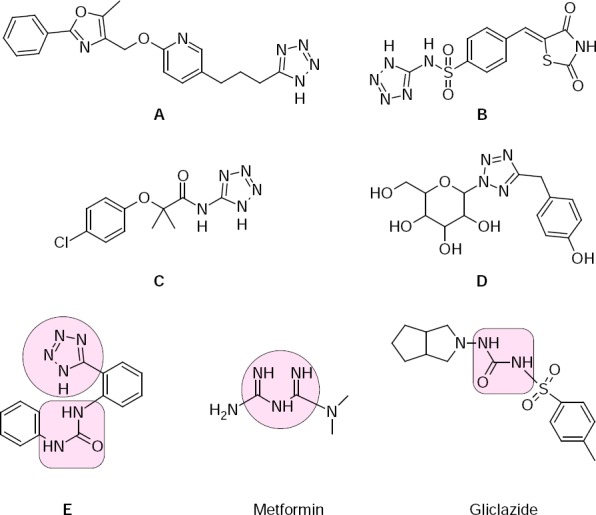
Structures of tetrazolo-containing antidiabetic drug-like structures and drugs.

Some efforts for this approach were made by Yu Momose and coauthors, with their 5-(4-alkoxyphenylalkyl)-1*H*-tetrazoles, with the best one being **A**, which showed potent glucose- and lipid-lowering activities in KKAy mice (Fig. 1) [1].

The antidiabetic effects of those compounds were considered to be due to their potent agonistic activity for peroxisome proliferator-activated receptor. Also Pattan S. R. et al. have obtained 2,4-thiazolidinedione derivatives **B**, containing a tetrazole ring for their antidiabetic activity [2]. Most of the substances showed good activity, compared with Glibenclamide. Navarrete-Vázquez G. et al. have synthesized a tetrazole isosteric analogue of clofibric acid as a potent hypoglycemic agent, compound **C** [3]. Besides docking studies, synthesis and *in* *vivo* studies of hypoglycemic activity among tetrazole-bearing *N*-glycosides (**D**) as sodium-glucose co-transporter 2 (SGLT2) inhibitors were made by Kumari B. et al. and Y L Gao et al. (Fig. 1) [4, 5].

Derivatives of quinazoline have also possessed antidiabetic activity. Such derivatives, 2-*sec*-amino-3*H*-quinazolin-4-ones, displayed a significant reduction in blood glucose level in streptozotocin and sucrose-loaded rat models [6] as well as 1-thioxo-1,2,7,8,9,10-hexahydro-3*H*-pyrimido[1,6-*a*]quinazolin-3-one [7]. Also, *N*-substituted-(4-oxo-2-substituted-phenylquinazolin-3-(4*H*)-yl), namely *N*-[7-chloro-2-(4-methoxyphenyl)-4-oxoquin-azolin-3(4*H*)-yl]-4 nitrobenzenesulfonamide, exhibited high antidiabetic potential [8]. In addition, *N*-4(substituted phenyl)-5-{[(2-phenylquinazolin-4-yl)-oxy]-methyl}-1,3,4-thiadiazol-2-amine derivatives according to docking glide score showed a high potency with regards to hypoglycemic activity [9].

Hence, the aim of the work was to synthesize substances which combine two different structural fragments of antidiabetic drugs (sulfonylureas and biguanides). As it is highlighted in Figure 1 by oval and rectangular marks, structure **E** has both fragments: urea and tetrazole, which is similar to the biguanidine moiety. So, to achieve our goal, a novel series of 1-[2-(1*H*-tetrazol-5-yl)-R^1^-phenyl]-3-R^2^-phenyl(ethyl)ureas (**E**) were synthesized and their hypoglycemic effect was investigated.

## Results and Discussion

### Chemistry

The R^1^-2-(1*H*-tetrazol-5-yl)anilines (**1.1**–**1.5**) were used as starting compounds and were obtained by known methods, described elsewhere [10–15]. Treatment of R^1^-2-(1*H*-tetrazol-5-yl)-phenylamines with R^2^-phenylisocyanates was carried out under different conditions: heating with reflux in glacial acetic acid or stirring at room temperature also in acetic medium or alternatively in dioxane (Scheme 1). In the first case, cyclization with formation of tetrazolo[1,5-*c*]quinazolin-5(6*H*)-ones (**3.1–3.5**) was observed, while 1-[2-(1*H*-tetrazol-5-yl)-R^1^-phenyl]-3-R^2^-phenylureas (**2.6–2.31**) were synthesized by the second approach. It was established that ethyl isocyanate yields and purity were higher in acetic acid compared to dioxane medium. That is why acetic acid medium was chosen as the major one. Interaction of ethyl isocyanate with R_1_-2-(1*H*-tetrazol-5-yl)anilines was also held at room temperature in glacial acetic acid with stirring and resulted in 1-[2-(1*H*-tetrazol-5-yl)phenyl]-3-ethylureas (**2.1–2.5**).

**Sch. 1:**
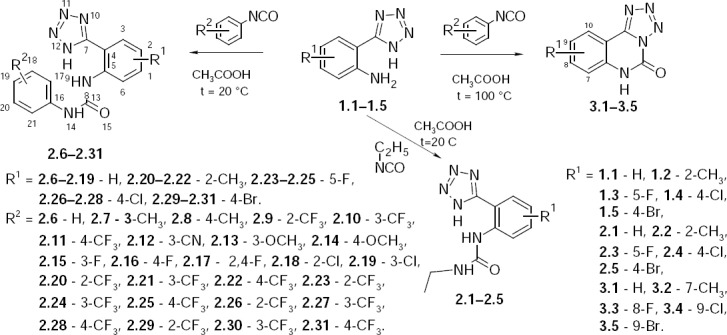
Synthesis of 1-[2-(1*H*-tetrazol-5-yl)-R^1^-phenyl]-3-R^2^-phenyl(ethyl)ureas (**2.1–2.31**) and R^1^-tetrazolo[1,5-*c*]quinazolin-5(6*H*)-ones (**3.1–3.5**).

The identity of the synthesized compounds was confirmed by IR, LC-, EI-MS, ^1^H-NMR, and elemental analysis. LC-MS of the synthesized compounds in “soft” ionization (chemical ionization at atmospheric pressure) allowed us to register the molecular ion peak [M+1] in high intensity.

Signals for the aromatic quinazoline protons and NH group were observed in the ^1^H-NMR spectra of the R^1^-tetrazolo[1,5-*c*]quinazolin-5(6*H*)-ones (**3.1–3.5**) in accordance with the substituents. The NH group was registered at 10.11–12.80 ppm, H-7 at 7.50–7.20 ppm, H 8 at 7.81–7.33 ppm, H-9 at 7.71–7.33 ppm, and H-10 at 8.36–8.12 ppm.

Three NH groups of 1-[2-(1*H*-tetrazol-5-yl)-R^1^-phenyl]-3-R^2^-phenyl(ethyl)ureas **2.1–2.31** were also detected. The NH groups of the uric fragment were closely detected as two one-proton singlets, H-9 at 10.89–8.14 ppm, and H-14 at 10.14–7.18 ppm (atom numeration according to Scheme 1). While the H-8 group of tetrazol was registered in a very weak field as a low-intensity broad singlet at 16.85–16.18 ppm, this signal was not observed for compounds **2.7**–**2.9**, **2.11**, **2.24**, and **2.25**. Aromatic protons of both phenyls were detected in the range of 8.42–6.47 ppm, and their multiplicity and intensity corresponded to the structures. Signals in the strong field corresponded to the alkyl moieties of 1-[2-(1*H*-tetrazol-5-yl)-R^1^-phenyl]-3-R^2^-ethylureas **2.1–2.5**.

A group of weak bands at the region of 1746–1587 cm^−1^ (overtone and component frequencies) were registered for all compounds due to the aromatic moiety, and the number and position of substituents were determined in the benzene ring. Bending vibrations of aromatic *ν*_CH_ were registered at the range of 3086–3047 cm^−1^. Also registered were out-of-plane bending vibrations at 999–659 cm^−1^ and in-plane vibrations at 1236–951 cm^−1^. The uric fragment in the IR-spectra was characterized by deformation vibrations of the band N-H and C-N («Amide II») at 1690–1649 cm^−1^.

### Pharmacology

#### Hypoglycemic Activity

*Molecular Docking*. To reveal the possible interaction, molecular docking studies were conducted. As the targets, it was decided to use the following ones: 11β-hydroxysteroid dehydrogenase 1 (HSD11B1, PDB ID - 3QQP), γ-peroxisome proliferator-activated receptor (γ-PPAR, PDB ID - 2XKW), and dipeptidyl peptidase-4 (DPP4, PDB ID - 2RGU) [15]. The mentioned targets were selected as the main and probable enzymes that are responsible for the hypoglycemic activity. Pioglitazone, Linagliptin, and (4-(1*H*-imidazol-4-yl)piperidin-1-yl)(3,4-dihydroquinolin-1(2*H*)-yl)methanone were used as a reference. These structures were obtained from pdb-files of proteins and redocked in order to have a value of affinity, which can be used as a reference (Table 1). It should be noted that compound **2.11** demonstrated the best affinity to both γ-PPAR and DPP4 with −10.1 kcal/mol and −9.9 kcal/mol, while substance **2.29** fit 11β-hydroxysteroid dehydrogenase 1 with the best value of −9.9 kcal/mol.

**Tab. 1:**
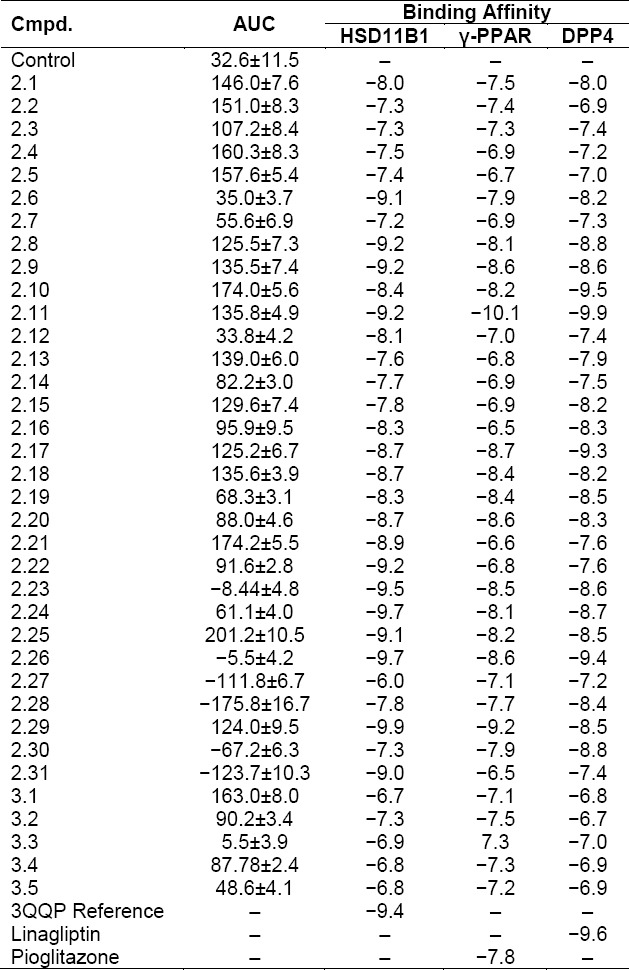
Dynamic AUC of 1-[2-(1*H*-tetrazol-5-yl)-R^1^-phenyl]-3-R^2^-phenyl(ethyl)ureas and R^1^-tetrazolo[1,5-*c*]quinazolin-5(6*H*)-ones and affinity of the synthesized compounds according to docking studies, kcal/mol

**Fig. 2:**
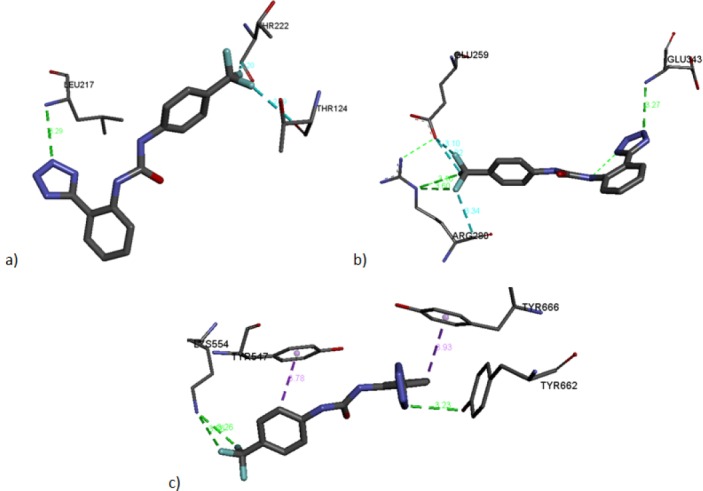
Interaction between compound **2.11** with HSD11B11 (a), with γ-PPAR (b), and with DPP4 (c). Green - classical hydrogen bonds, purple - hydrophobic pi-sigma interactions, light blue - carbon-bound fluorine interactions.

Visualization of the interactions for compound **2.11** is presented in Fig. 2. With HSD11B1 according to the docking study, such interactions were observed: the CF_3_ group has carbon-bound fluorine interactions between both THR222 and THR124, and a classical hydrogen bond between LEU217 and N of tetrazol (Fig. 2, a). With γ-PPAR, the CF_3_ group of compound **2.11** has both carbon-bound fluorine interactions and a classical hydrogen bond between GLU259 and ARG280, as well as a classical hydrogen bond between GLU343 and N of tetrazol (Fig. 2, b). Speaking of DPP4: a classical hydrogen bond between N of tetrazol and TYR662, two classical hydrogen bonds between CF_3_ and LYS554, and two hydrophobic pi-sigma interactions between TYR547 and the second carbon of CF_3_Ph and between TYR666 and the sixth carbon of Ph (Fig. 2, c). So it can be noted that the classical hydrogen bond between the N of tetrazol is present for all proteins.

The results, obtained by preliminary experimental screening, were interpreted by the area under the curve (AUC), Table 1. Higher values of AUC represent the more expressed hypoglycemic action, while a negative value means an increase in glucose concentration. According to the dynamic area under the curve, most of the compounds revealed their ability to reduce blood glucose level. Among the most active substances turned out to be **2.1**, **2.2**, **2.9**, **2.10**, **2.25**, **3.1** with ethyl and CF_3_ fragments. At the same time, introducing chloro- (**2.27**, **2.28**), bromo- (**2.30**, **2.31**), fluoro- (**3.3**), and methyl- (**2.21**) substituents revealed the glucose-increasing effect during the experiment. More expressed action was registered on the 4 and 6 h after administration that can be caused by the time of absorption in the digestive tract.

Based on the results of preliminary screening and docking studies, the compounds were selected for the study of specific hypoglycemic activity on Dexamethasone diabetes models, namely **2.1**, **2.2**, **2.4**, **2.9–2.11**, **2.21**, **3.1.**

On the next stage, the hypoglycemic action of compounds **2.1**, **2.2**, **2.4**, **2.9–2.11**, **2.21**, **3.1** were studied using glucocorticoid-induced insulin resistance models. Moderate basal hyperglycemia was caused by subcutaneous injection of Dexamethasone (0.125 mg/kg) to rats for 15 days. Glucose homeostasis characteristics were conducted in terms of basal glycemia, insulinemia, and tolerance to carbohydrates which were determined by the following tests.

#### Oral Glucose Tolerance Test

Glucose (2 g/kg) was intragastrically administrated via noninvasive probe. Blood samples for glucose analysis were collected before administration and after 15, 30, 60, and 120 min (Table 2).

**Tab. 2:**
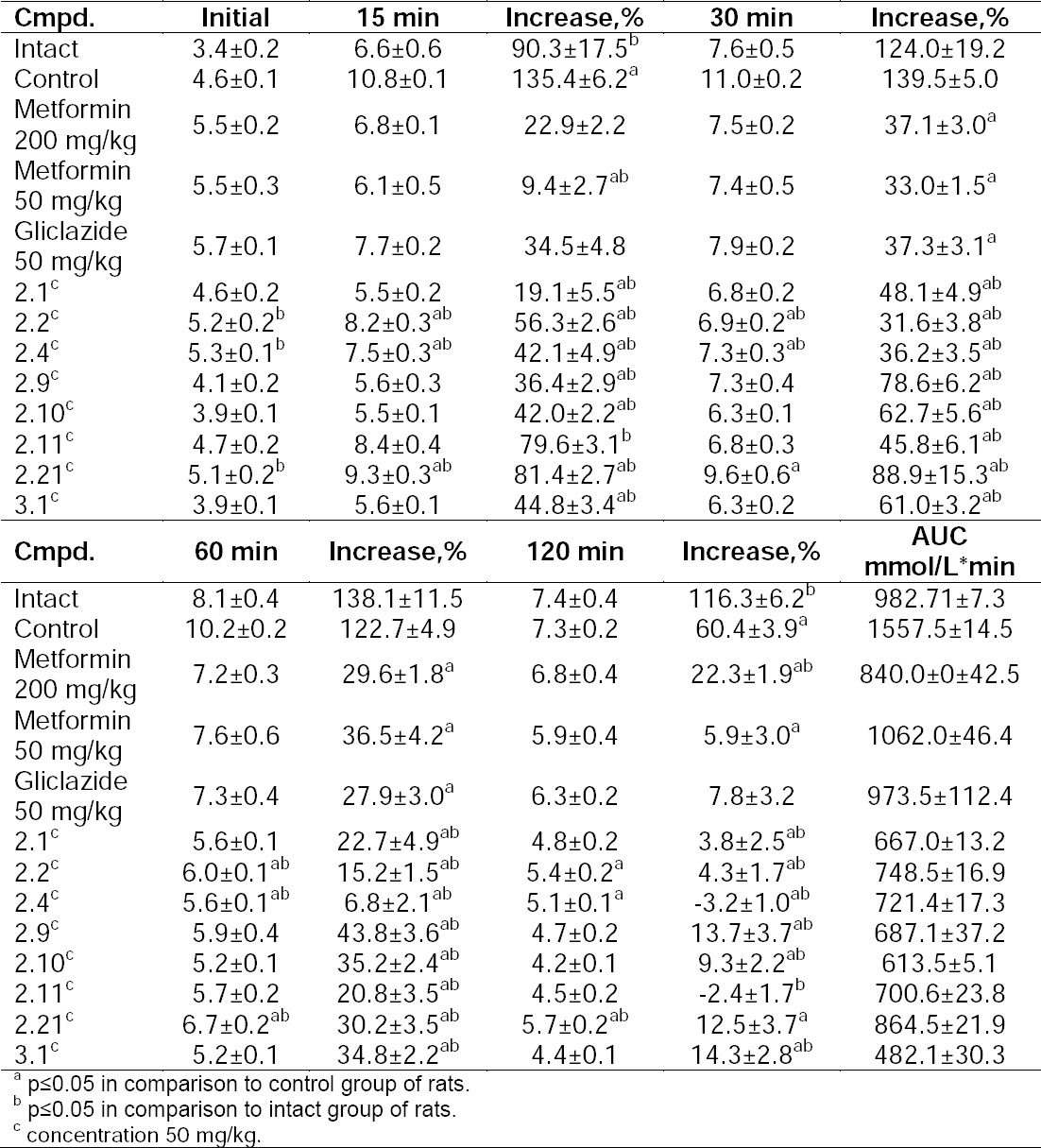
Levels of glucose after oral test for glucose tolerance, mmol/L

Evaluation of specific hypoglycemic action (oral test for glucose tolerance) in the condition of glucocorticoid-induced insulin resistance showed that all compounds revealed a glucose-lowering effect. For such compounds **2.1** and **3.1**, the AUC was two times lower, compared to the reference drugs «Metformin» (50 and 200 mg/kg) and «Gliclazide» (50 mg/kg, Table 2).

#### Rapid Insulin Test

This test can evaluate the sensitivity of both the liver and peripheral tissues to insulin action, taking into account the inhibition of glucose production in the liver and increase in muscle glucose utilization due to the effect of the hormone. Sensitivity to insulin can be determined by calculating the percentage of basal glucose decrease after 30 min of intraperitoneal injection of the hormone into fasting animals (1 unit/kg, Table 3).

**Tab. 3:**
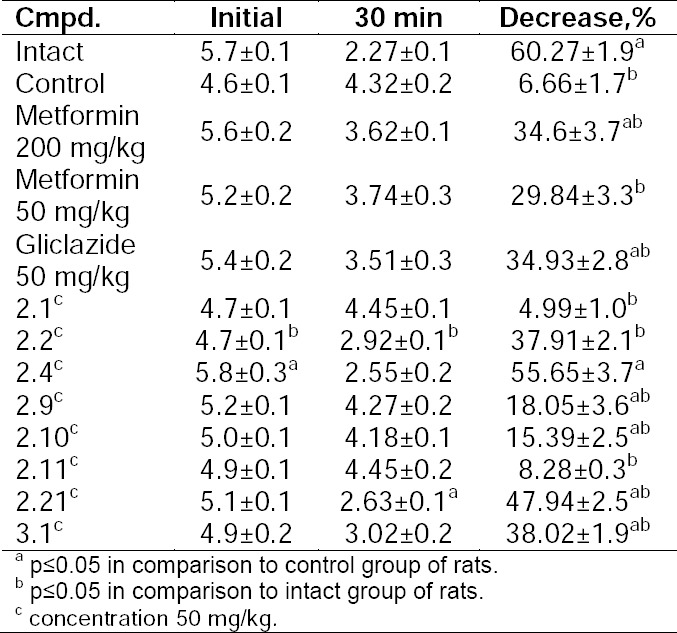
Levels of glucose after the rapid insulin test, mmol/L

The rapid insulin test with glucocorticoid-induced insulin resistance showed that compounds **2.2**, **2.4**, **2.21**, **3.1** were the most active, and **2.4** and **2.21** even exceeded the reference drugs.

#### Adrenalin Test

0.18% adrenaline solution at a dose of 0.5 mg/kg was administered to rats for determination of the sensitivity to corticosteroids. Blood glucose levels were tested before the introduction of adrenaline and after 30 and 90 min. Results can be seen in Table 4.

**Tab. 4:**
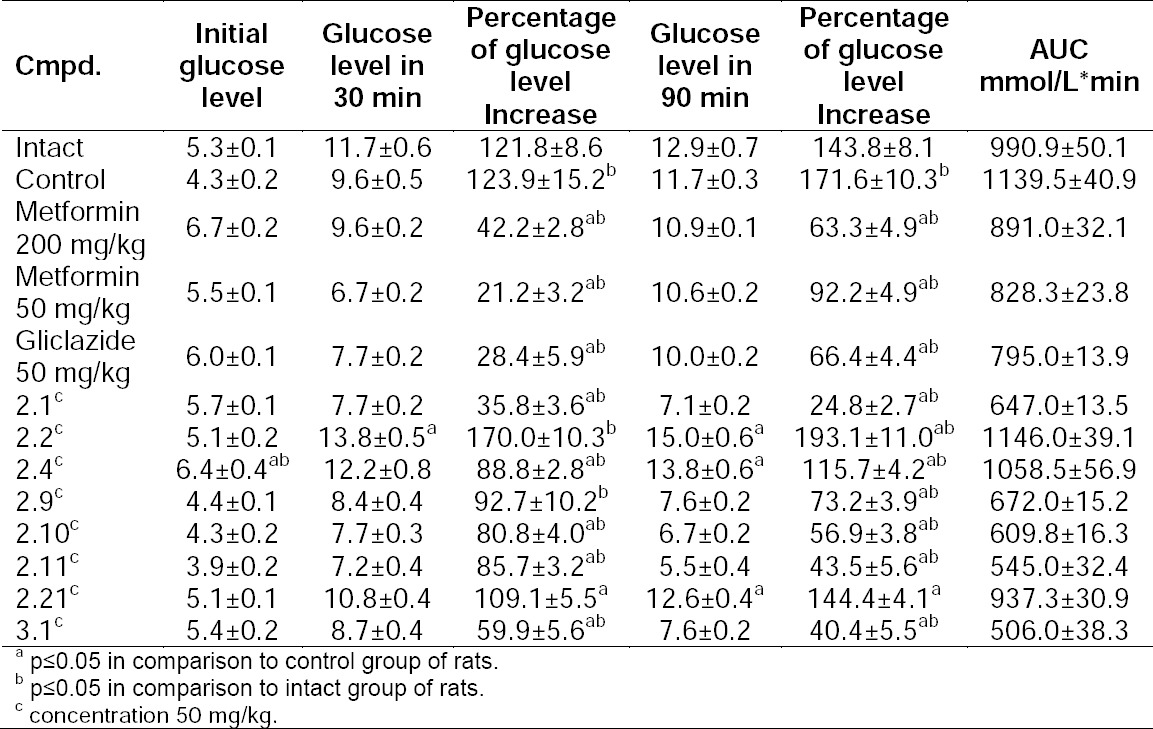
Levels of glucose after the adrenaline test, mmol/L

According to the adrenaline test, compounds **2.11** and **3.1** showed high hypoglycemic activity, higher than the references Metformin and Gliclazide. Thus, preliminary screening and Dexamethasone diabetes models proved the high hypoglycemic activity of the synthesized compounds. Additionally, the molecular docking study showed a possible interaction with the receptor.

## Conclusion

A range of 1-[2-(1*H*-tetrazol-5-yl)-R^1^-phenyl]-3-R^2^-phenyl(ethyl)ureas (**2.1–2.31**) and R^1^ tetrazolo[1,5-*c*]quinazolin-5(6*H*)-ones (**3.1–3.5**) combining two different structural fragments of antidiabetic drugs, namely sulfonylureas and biguanides, were obtained. Reaction conditions and appropriate products were distinguished. The docking study showed that the obtained group of compounds were prospective, and showed good affinity to γ-PPAR, DPP4, and 11β-hydroxysteroid dehydrogenase 1. It was found by docking classical hydrogen bonds between tetrazol and proteins, the hydrophobic pi-sigma interactions were crucial in the formation of the interaction. The best affinity appeared to be for compound **2.11** with both γ-PPAR and DPP4. Purposeful synthesis based on the docking studies revealed a new class of hypoglycemic compounds. The Dexamethasone diabetes models were used to determine the specific hypoglycemic activity for eight compounds. Substances **2.4**, **2.21**, **3.1** revealed high activity, in some cases exceeding the reference drugs Metformin (50 and 200 mg/kg) and Gliclazide (50 mg/kg). Investigation of the activity mechanism is the next step of the study. So, the substances library enlargement among the substituted 1-[2-(1*H*-tetrazol-5-yl)-R^1^-phenyl]-3-R^2^-phenyl(ethyl)ureas will be used to improve their hypoglycemic activity.

## Experimental

### Chemistry

Melting points were determined in open capillary tubes in a «Stuart SMP30» apparatus and were uncorrected. The elemental analyses (C, H, N, O) were performed using the ELEMENTAR vario EL cube analyzer. IR spectra (4000–600 cm^−1^) were recorded on a Bruker ALPHA FT-IR spectrometer using a module ATR eco ZnSe. ^1^H-NMR spectra (400 MHz) were recorded on a Varian-Mercury 400 (Varian Inc., Palo Alto, CA, USA) spectrometer with TMS as internal standard in DMSO-d_6_ solution. LC-MS were recorded using a chromatography / mass spectrometric system which consisted of high-performance liquid chromatography «Agilent 1100 Series» (Agilent, Palo Alto, CA, USA) equipped with a diode-matrix and mass-selective detector «Agilent LC/MSD SL» (atmospheric pressure chemical ionization – APCI).

Substances **1.1–1.5** were synthesized according to the reported procedures [10, 11]. Other starting materials and solvents were obtained from commercially available sources and used without additional purification.

### General Procedure for the Synthesis of 1-[2-(1H-tetrazol-5-yl)-R^1^-phenyl]-3-R^2^-phenyl(ethyl)ureas (2.1–2.31)

After 1.0 g of 2-(1*H*-tetrazol-5-yl)aniline (0.0062 mol) was dissolved in acetic acid, 0.74 g of isocyanatobenzene (0.0062 mol) was added. The mixture was stirred at room temperature for 5 h. After cooling, the formed precipitate was filtered, washed with water, dried, and crystallized from methanol.

#### 1-(2-(1H-Tetrazol-5-yl)phenyl)-3-ethylurea (**2.1**)

White crystals, yield: 33%; mp: 174–176°C; IR (cm^−1^): 3331, 3118, 3046, 2969, 2915, 2873, 2756, 2675, 2631, 2603, 2570, 1659, 1651, 1619, 1586, 1548, 1537, 1530, 1484, 1454, 1434, 1377, 1358, 1311, 1285, 1259, 1241, 1167, 1151, 1138, 1103, 1070, 1051, 1020, 988, 947, 912, 879, 859, 838, 811, 771, 746, 732, 716, 700, 684, 665, 614. ^1^H-NMR: δ (ppm): 16.68 (br.s, 1H, 8), 9.65 (s, 1H, 9), 8.40 (d, *J* = 8.4 Hz, 1H, 3), 7.81 (d, *J* = 7.5 Hz, 1H, 6), 7.39 (t, *J* = 7.8 Hz, 1H, 1), 7.18 (br.s, 1H, 14), 7.03 (t, *J* = 7.4 Hz, 1H, 2), 3.20–3.07 (m, 2H, CH_2_), 1.12 (t, *J* = 7.0 Hz, 3H, CH_3_). LC-MS: m/z = 233 [M + H]^+^. Anal. Calcd. for C_10_H_12_N_6_O: C, 51.72; H, 5.21; N, 36.19; O, 6.89. Found: C, 51.79; H, 5.12; N, 36.25; O, 6.81.

#### 1-Ethyl-3-(2-methyl-6-(1H-tetrazol-5-yl)phenyl)urea (**2.2**)

White crystals, yield: 61%; mp: 186–188°C; IR (cm^−1^): 3332, 2974, 2922, 2890, 2864, 2835, 2690, 2584, 2531, 2445, 2360, 1840, 1641, 1612, 1592, 1556, 1503, 1479, 1463, 1407, 1383, 1271, 1253, 1233, 1198, 1164, 1096, 1053, 993, 956, 858, 799, 753, 711, 658, 607. ^1^H-NMR: δ (ppm): 16.28 (br.s, 1H, 8), 8.14 (s, 1H, 9), 7.53 (d, *J* = 6.3 Hz, 1H, 3), 7.33 (d, *J* = 6.8 Hz, 1H, 1), 7.19 (t, *J* = 7.1 Hz, 1H, 2), 6.49 (br.s, 1H, 14), 3.02 (d, *J* = 6.2 Hz, 2H, CH_2_), 2.28 (s, 3H, PhCH_3_), 1.04 (t, *J* = 6.7 Hz, 3H, CH_3_). LC-MS: *m/z* = 247 [M + H]^+^. Anal. Calcd. for C_11_H_14_N_6_O: C, 53.65; H, 5.73; N, 34.13; O, 6.50. Found: C, 53.53; H, 5.80; N, 34.03; O, 6.59.

#### 1-Ethyl-3-(5-fluoro-2-(1H-tetrazol-5-yl)phenyl)urea (**2.3**)

Beige crystals, yield: 54%; mp: 190–192°C; IR (cm^−1^): 3124, 3069, 2989, 2928, 2780, 2705, 2633, 2597, 1678, 1603, 1554, 1504, 1480, 1442, 1392, 1370, 1304, 1291, 1267, 1242, 1168, 1143, 1113, 1085, 1060, 1023, 1012, 998, 979, 956, 872, 832, 802, 764, 692, 670, 630, 605. ^1^H-NMR: δ (ppm): 16.82 (br.s, 1H, 8), 11.17 (s, 1H, 9), 8.39 (d, *J* = 11.9 Hz, 1H, 6), 7.97 (t, *J* = 7.1 Hz, 1H, 3), 6.95 (t, *J* = 6.9 Hz, 1H, 2), 2.5 (s, 2H, CH_2_), 2.23 (s, 3H, CH_3_). Anal. Calcd. for C_10_H_11_FN_6_O: C, 48.00; H, 4.43; N, 33.58; O, 6.39. Found: C, 48.09; H, 4.31; N, 33.69; O, 6.29.

#### 1-(4-Chloro-2-(1H-tetrazol-5-yl)phenyl)-3-ethylurea (**2.4**)

White crystals, yield: 53%; mp: 210-212°C; IR (cm^−1^): 3125, 3074, 2953, 2910, 2835, 2787, 1677, 1620, 1591, 1536, 1486, 1417, 1375, 1313, 1262, 1156, 1128, 1112, 1099, 1078, 1059, 1016, 1008, 962, 883, 849, 826, 759, 689, 672, 613. ^1^H-NMR: δ (ppm): 16.84 (br.s, 1H, 8), 10.89 (s, 1H, 9), 8.52 (d, *J* = 9.0 Hz, 1H, 6), 8.01 (s, 1H, 3), 7.44 (d, *J* = 8.7 Hz, 1H, 1), 2.5 (s, 2H, CH_2_), 2.20 (s, 3H, CH_3_). LC-MS: m/z = 238 [M + H]^+^. Anal. Calcd. for C_10_H_11_ClN_6_O: C, 45.04; H, 4.16; N, 31.51; O, 6.00. Found: C, 45.16; H, 4.02; N, 31.55; O, 6.01.

#### 1-(4-Bromo-2-(1H-tetrazol-5-yl)phenyl)-3-ethylurea (**2.5**)

Beige crystals, yield: 61%; mp: 205–207°C; IR (cm^−1^): 3150, 3114, 3070, 2943, 2910, 2829, 2784, 1676, 1650, 1618, 1587, 1531, 1484, 1416, 1372, 1337, 1311, 1266, 1155, 1106, 1077, 1060, 1006, 959, 883, 826, 767, 740, 689, 668. ^1^H-NMR: δ (ppm): 16.83 (br.s, 1H, 8), 10.90 (s, 1H, 9), 8.46 (d, *J* = 8.9 Hz, 1H, 6), 8.14 (s, 1H, 3), 7.57 (d, *J* = 8.9 Hz, 1H, 1), 2.5 (s, 2H, CH_2_), 2.20 (s, 3H, CH_3_). LC-MS: m/z = 284 [M + H]^+^. Anal. Calcd. for C_10_H_11_BrN_6_O: C, 38.60; H, 3.56; N, 27.01; O, 5.14. Found: C, 38.73; H, 3.42; N, 27.17; O, 5.01.

#### 1-(2-(1H-Tetrazol-5-yl)phenyl)-3-phenylurea (**2.6**)

Beige crystals, yield: 70%; mp: 176–178°C; IR (cm^−1^): 3298, 3075, 2879, 2737, 1663, 1654, 1616, 1607, 1594, 1557, 1541, 1533, 1520, 1506, 1497, 1487, 1438, 1405, 1320, 1295, 1240, 1177, 1155, 1113, 1102, 1077, 1051, 1039, 1027, 996, 958, 919, 896, 883, 873, 847, 830, 821, 795, 777, 756, 740, 723, 703, 680, 659, 645, 634, 620. ^1^H-NMR: δ (ppm): 16.69 (br.s, 1H, 8), 9.80 (s, 1H, 9), 9.60 (s, 1H, 14), 8.34 (d, *J* = 8.3 Hz, 1H, 6), 7.85 (d, *J* = 6.5 Hz, 1H, 3), 7.52 (d, *J* = 7.7 Hz, 2H, 17, 21), 7.46 (t, *J* = 7.6, Hz 1H, 1), 7.22 (t, *J* = 7.4 Hz, 2H, 18, 20), 7.12 (t, *J* = 7.3 Hz, 1H, 2), 6.92 (t, *J* = 6.9 Hz, 1H, 19). LC-MS: *m/z* = 281 [M + H]^+^. Anal. Calcd. for C_14_H_12_N_6_O: C, 59.99; H, 4.32 N, 29.98; O, 5.71. Found: C, 59.94; H, 4.36 N, 29.93; O, 5.76.

#### 1-(2-(1H-Tetrazol-5-yl)phenyl)-3-(m-tolyl)urea (**2.7**)

White crystals, yield: 39%; mp: 158–160°C; IR (cm^−1^): 3283, 3118, 3064, 2917, 2762, 1754, 1688, 1680, 1666, 1658, 1649, 1613, 1592, 1546, 1502, 1482, 1450, 1424, 1379, 1297, 1236, 1155, 1116, 1068, 1051, 995, 958, 934, 901, 861, 772, 743, 704, 692, 668, 638, 614, 606. ^1^H-NMR: δ (ppm): 9.77 (s, 1H, 9), 9.51 (s, 1H, 14), 8.34 (d, *J* = 8.3 Hz, 1H, 6), 7.85 (d, *J* = 7.4 Hz, 1H, 3), 7.45 (t, *J* = 7.4 Hz, 1H, 1), 7.35 (s, 1H, 17), 7.28 (d, *J* = 7.5 Hz, 1H, 21), 7.10 (m, 2H, 2, 20), 6.73 (d, *J* = 7.0 Hz, 1H, 19), 2.32 (s, 2H, CH_3_). LC-MS: 295 *m/z* = [M + H]^+^. Anal. Calcd. for C_15_H_14_N_6_O: C, 61.21; H, 4.79; N, 28.55; O, 5.44. Found: C, 61.27; H, 4.74; N, 28.59; O, 5.37.

#### 1-(2-(1H-Tetrazol-5-yl)phenyl)-3-p-tolylurea (**2.8**)

White crystals, yield: 79%; mp: 160-164°C; IR (cm^−1^): 3311, 3052, 2781, 1660, 1618, 1588, 1538, 1514, 1486, 1446, 1403, 1307, 1292, 1238, 1208, 1154, 1112, 1073, 1048, 1017, 996, 951, 938, 919, 893, 859, 821, 791, 772, 744, 720, 702, 674, 659, 646, 636, 612. ^1^H-NMR: δ (ppm): 9.78 (s, 1H, 9), 9.48 (s, 1H, 14), 8.34 (d, *J* = 8.3 Hz, 1H, 6), 7.85 (d, *J* = 7.6 Hz, 1H, 3), 7.44 (t, *J* = 7.7 Hz, 1H, 1), 7.38 (d, *J* = 7.6 Hz, 2H, 17, 21), 7.11 (t, *J* = 7.4 Hz, 1H, 2), 7.02 (d, *J* = 7.6 Hz, 2H, 18, 20), 2.28 (s, 3H, CH_3_). LC-MS: *m/z* = 295 [M + H]^+^. Anal. Calcd. for C_15_H_14_N_6_O: C, 61.21; H, 4.79; N, 28.55; O, 5.44. Found: C, 61.25; H, 4.74; N, 28.57; O, 5.41.

#### 1-(2-(1H-Tetrazol-5-yl)phenyl)-3-(2-(trifluoromethyl)phenyl)urea (**2.9**)

White crystals, yield: 70%; mp: 265–267°C; IR (cm^−1^): 3320, 1650, 1613, 1592, 1543, 1504, 1494, 1453, 1405, 1319, 1290, 1269, 1256, 1227, 1171, 1116, 1086, 1060, 1032, 1015, 991, 962, 908, 771, 746, 736, 711, 680, 655, 634, 606. ^1^H-NMR: δ (ppm): 9.71 (s, 1H, 9), 8.83 (s, 1H, 14), 8.18 (d, *J* = 6.9 Hz, 1H, 6), 7.77 (dd, *J* = 18.1, 7.1 Hz, 2H, 1, 3), 7.62 (d, *J* = 6.9 Hz, 1H, 21), 7.57 (t, *J* = 7.6 Hz, 1H, 2), 7.45 (br.s, 1H, 18), 7.28 (br.s, 1H, 20), 7.16 (br.s, 1H, 19). LC-MS: 349 *m/z* = [M + H]^+^. Anal. Calcd. for C_15_H_11_F_3_N_6_O: C, 51.73; H, 3.18; N, 24.13; O, 4.59. Found: C, 51.76; H, 3.15; N, 24.16; O, 4.54.

#### 1-(2-(1H-Tetrazol-5-yl)phenyl)-3-(3-(trifluoromethyl)phenyl)urea (**2.10**)

White crystals, yield: 74%; mp: 200–202°C; IR (cm^−1^): 3284, 3116, 2959, 2890, 2852, 2816, 2732, 2615, 1664, 1613, 1554, 1529, 1489, 1453, 1442, 1389, 1372, 1333, 1306, 1254, 1242, 1182, 1170, 1157, 1134, 1111, 1078, 1042, 1000, 958, 937, 924, 886, 856, 805, 786, 775, 756, 739, 708, 694, 681, 658, 647, 626. ^1^H-NMR: δ (ppm): 16.85 (br.s, 1H, 8), 9.99 (s, 2H, 9, 14), 8.38 (d, *J* = 8.2 Hz, 1H, 6), 7.98 (s, 1H, 17), 7.88 (d, *J* = 7.3 Hz, 1H, 3), 7.74 (d, *J* = 7.8 Hz, 1H, 21), 7.47 (t, *J* = 7.5 Hz, 1H, 1), 7.41 (t, *J* = 7.6 Hz, 1H, 20), 7.20 (d, *J* = 7.3 Hz, 1H, 19), 7.15 (t, *J* = 7.2 Hz, 1H, 2). LC-MS: 349 *m/z* = [M + H]^+^. Anal. Calcd. for C_15_H_11_F_3_N_6_O: C, 51.73; H, 3.18; N, 24.13; O, 4.59. Found: C, 51.84; H, 3.11; N, 24.23; O, 4.65.

#### 1-(2-(1H-Tetrazol-5-yl)phenyl)-3-(4-(trifluoromethyl)phenyl)urea (**2.11**)

White crystals, yield: 65%; mp: 224–246°C; IR (cm^−1^): 3321, 3061, 2921, 2850, 1723, 1692, 1667, 1619, 1591, 1563, 1537, 1487, 1454, 1414, 1320, 1301, 1281, 1237, 1169, 1112, 1080, 1065, 1031, 1016, 991, 954, 857, 838, 822, 778, 754, 722, 705, 687, 674, 651, 625. ^1^H-NMR: δ (ppm): 10.01 (s, 1H, 9), 9.96 (s, 1H, 14), 8.35 (d, *J* = 8.3 Hz, 1H, 6), 7.88 (d, *J* = 5.9 Hz, 1H, 3), 7.73 (m, 3H, 1, 17, 21), 7.48 (m, 2H, 18, 20), 7.14 (t, *J* = 7.1 Hz, 1H, 2). Anal. Calcd. for C_15_H_11_F_3_N_6_O: C, 51.73; H, 3.18; N, 24.13; O, 4.59. Found: C, 51.79; H, 3.04; N, 24.21; O, 4.51.

#### 1-(2-(1H-Tetrazol-5-yl)phenyl)-3-(3-cyanophenyl)urea (**2.12**)

White crystals, yield: 68%; mp: 224–226°C; IR (cm^−1^): 3303, 3275, 3123, 2924, 2863, 2759, 2313, 2238, 1712, 1690, 1666, 1650, 1642, 1615, 1590, 1555, 1513, 1477, 1461, 1445, 1423, 1397, 1376, 1331, 1303, 1294, 1262, 1232, 1174, 1153, 1089, 1071, 1048, 1008, 996, 960, 950, 905, 888, 853, 810, 789, 773, 744, 703, 673, 657, 637. ^1^H-NMR: δ (ppm): 16.84 (br.s, 1H, 8), 10.01 (s, 2H, 9, 14), 8.36 (d, *J* = 8.4 Hz, 1H, 6), 8.01 (s, 1H, 17), 7.88 (d, *J* = 7.6 Hz, 1H, 3), 7.77 (d, *J* = 8.0 Hz, 1H, 21), 7.47 (t, *J* = 7.7 Hz, 1H, 1), 7.40 (t, *J* = 7.8 Hz, 1H, 20), 7.27 (d, *J* = 7.1 Hz, 1H, 19), 7.15 (t, *J* = 7.4 Hz, 1H, 2). LC-MS: *m/z* = 306 [M + H]^+^. Anal. Calcd. for C_15_H_11_N_7_O: C, 59.01; H, 3.63; N, 32.12; O, 5.24. Found: C, 59.05; H, 3.61; N, 32.15; O, 5.22.

#### 1-(2-(1H-Tetrazol-5-yl)phenyl)-3-(3-methoxyphenyl)urea (**2.13**)

White crystals, yield: 67%; mp: 175–177°C; IR (cm^−1^): 3353, 3286, 2959, 2927, 2853, 1724, 1712, 1690, 1650, 1604, 1550, 1511, 1496, 1461, 1449, 1436, 1417, 1403, 1325, 1295, 1283, 1264, 1245, 1223, 1203, 1182, 1152, 1108, 1069, 1047, 1030, 992, 922, 861, 842, 768, 757, 741, 707, 680, 625, 605. ^1^H-NMR: δ (ppm): 16.73 (br.s, 1H, 8), 9.79 (s, 1H, 9), 9.60 (s, 1H, 14), 8.34 (d, *J* = 8.4 Hz, 1H, 6), 7.85 (d, *J* = 7.5 Hz, 1H, 3), 7.46 (t, *J* = 7.6 Hz, 1H, 1), 7.23 (s, 1H, 17), 7.11 (m, 2H, 2, 21), 7.03 (d, *J* = 7.8 Hz, 1H, 20), 6.47 (d, *J* = 7.3 Hz, 1H, 19), 3.76 (s, 3H, CH_3_). LC-MS: 311 *m/z* = [M + H]^+^. Anal. Calcd. for C_15_H_14_N_6_O_2_: C, 58.06; H, 4.55; N, 27.08; O, 10.31. Found: C, 58.17; H, 4.41; N, 27.27; O, 10.21.

#### 1-(2-(1H-Tetrazol-5-yl)phenyl)-3-(4-methoxyphenyl)urea (**2.14**)

White crystals, yield: 59%; mp: 170–172°C; IR (cm^−1^): 3284, 3265, 1672, 1615, 1599, 1539, 1511, 1487, 1443, 1412, 1300, 1274, 1228, 1171, 1108, 1060, 1027, 993, 946, 927, 824, 801, 761, 737, 707, 675, 649, 613. ^1^H-NMR: δ (ppm): 16.78 (br.s, 1H, 8), 9.78 (s, 1H, 9), 9.41 (s, 1H, 14), 8.35 (d, *J* = 8.4 Hz, 1H, 6), 7.84 (d, *J* = 7.5 Hz, 1H, 3), 7.84 (m, 3H, 1, 17, 21), 7.10 (t, *J* = 7.4 Hz, 1H, 2), 6.78 (d, *J* = 8.4 Hz, 2H, 18, 20), 3.75 (s, 3H, CH_3_). LC-MS: *m/z* = 311 [M + H]^+^. Anal. Calcd. for C_15_H_14_N_6_O_2_: C, 58.06; H, 4.55; N, 27.08; O, 10.31. Found: C, 58.17; H, 4.48; N, 27.19; O, 10.21.

#### 1-(2-(1H-Tetrazol-5-yl)phenyl)-3-(3-fluorophenyl)urea (**2.15**)

White crystals, yield: 80%; mp: 262–264°C; IR (cm^−1^): 3331, 3300, 3156, 3102, 3055, 2988, 2947, 2872, 1727, 1668, 1609, 1564, 1513, 1485, 1442, 1372, 1339, 1305, 1282, 1264, 1238, 1141, 1119, 1108, 1073, 1034, 1015, 998, 973, 891, 858, 805, 776, 761, 740, 721, 703, 681, 654. ^1^H-NMR: δ (ppm): 16.74 (br.s, 1H, 8), 9.88 (s, 1H, 9), 9.83 (s, 1H, 14), 8.34 (d, *J* = 8.4 Hz, 1H, 6), 7.87 (d, *J* = 7.3 Hz, 1H, 3), 7.48 (m, 2H, 1, 17), 7.21 (m, 2H, 21, 2), 7.14 (t, *J* = 7.4 Hz, 1H, 20), 6.64 (br.s, *J* = 7.6 Hz, 1H, 19). LC-MS: *m/z* = 299 [M + H]^+^. Anal. Calcd. for C_14_H_11_FN_6_O: C, 56.37; H, 3.72; N, 28.18; O, 5.36. Found: C, 56.46; H, 3.59; N, 28.16; O, 5.33.

#### 1-(2-(1H-Tetrazol-5-yl)phenyl)-3-(4-fluorophenyl)urea (**2.16**)

White crystals, yield: 62%; mp: 166–170°C; IR (cm^−1^): 3274, 2971, 2922, 1679, 1658, 1634, 1604, 1585, 1564, 1553, 1505, 1449, 1407, 1325, 1297, 1212, 1152, 1117, 1099, 1066, 1010, 990, 907, 844, 832, 799, 774, 754, 737, 702, 677, 648, 610. ^1^H-NMR: δ (ppm): 16.61 (br.s, 1H, 8), 9.85 (s, 1H, 9), 9.64 (s, 1H, 14), 8.34 (d, *J* = 8.5 Hz, 1H, 6), 7.86 (d, *J* = 7.5 Hz, 1H, 3), 7.52 (m, 2H, 17, 21), 7.45 (t, 1H, 1), 7.12 (t, *J* = 7.4 Hz, 1H, 2), 6.97 (m, 2H, 18, 20). LC-MS: *m/z* = 299 [M + H]^+^. Anal. Calcd. for C_14_H_11_FN_6_O: C, 56.37; H, 3.72; N, 28.18; O, 5.36. Found: C, 56.33; H, 3.76; N, 28.14; O, 5.39.

#### 1-(2-(1H-Tetrazol-5-yl)phenyl)-3-(2,4-difluorophenyl)urea (**2.17**)

White crystals, yield: 66%; mp: 268–270°C; IR (cm^−1^): 3284, 3117, 3063, 2973, 2923, 2851, 2761, 1744, 1693, 1650, 1619, 1591, 1549, 1504, 1486, 1447, 1429, 1303, 1253, 1231, 1202, 1169, 1153, 1141, 1099, 1061, 997, 962, 897, 849, 807, 788, 773, 741, 730, 696, 678, 668, 614. ^1^H-NMR: δ (ppm): 16.65 (br.s, 1H, 8), 9.62 (s, 1H, 9), 9.33 (s, 1H, 14), 8.22 (d, *J* = 8.0 Hz, 1H, 6), 7.96 (d, *J* = 5.7 Hz, 1H, 3), 7.79 (d, *J* = 6.2 Hz, 1H, 21), 7.46 (t, *J* = 6.7 Hz, 1H, 1), 7.14 (t, *J* = 7.1 Hz, 1H, 2), 6.98 (t, *J* = 9.6 Hz, 1H, 20), 6.89 (t, *J* = 7.0 Hz, 1H, 18). LC-MS: *m/z* = 317 [M + H]^+^. Anal. Calcd. for C_14_H_10_F_2_N_6_O: C, 53.17; H, 3.19; N, 26.57; O, 5.06. Found: C, 53.14; H, 3.22; N, 26.54; O, 5.08.

#### 1-(2-(1H-Tetrazol-5-yl)phenyl)-3-(2-chlorophenyl)urea (**2.18**)

White crystals, yield: 90%; mp: 253–255°C; IR (cm^−1^): 3333, 3299, 1865, 1642, 1618, 1585, 1539, 1502, 1470, 1440, 1416, 1294, 1214, 1175, 1130, 1112, 1099, 1052, 1032, 1020, 958, 877, 796, 776, 757, 747, 727, 710, 693, 671, 632, 612. ^1^H-NMR: δ (ppm): 16.65 (br.s, 1H, 8), 9.60 (s, 1H, 9), 8.91 (s, 1H, 14), 8.15 (d, *J* = 8.4 Hz, 1H, 6), 7.97 (d, *J* = 8.1 Hz, 1H, 3), 7.77 (d, *J* = 7.4 Hz, 1H, 21), 7.47 (t, *J* = 7.7 Hz, 1H, 1), 7.35 (d, *J* = 7.8 Hz, 1H, 18), 7.23 (t, *J* = 7.6 Hz, 1H, 20), 7.17 (t, *J* = 7.5 Hz, 1H, 2), 7.01 (t, *J* = 7.6 Hz, 1H, 19). LC-MS: *m/z* = 315 [M + H]^+^. Anal. Calcd. for C_14_H_11_ClN_6_O: C, 53.43; H, 3.52; N, 26.70; O, 5.08. Found: C, 53.49; H, 3.43; N, 26.71; O, 5.03.

#### 1-(2-(1H-Tetrazol-5-yl)phenyl)-3-(3-chlorophenyl)urea (**2.19**)

White crystals, yield: 56%; mp: 166–168°C; IR (cm^−1^): 3284, 2990, 2899, 2854, 2756, 1745, 1726, 1668, 1617, 1589, 1548, 1483, 1452, 1423, 1301, 1286, 1269, 1239, 1164, 1117, 1094, 1070, 997, 920, 873, 846, 802, 771, 742, 722, 710, 676, 635. ^1^H-NMR: δ (ppm): 16.81 (br.s 1H, 8), 9.91 (s, 1H, 9), 9.82 (s, 1H, 14), 8.35 (d, *J* = 8.4 Hz, 1H, 6), 7.87 (d, *J* = 7.7 Hz, 1H, 3), 7.71 (s, 1H, 17), 7.47 (t, *J* = 7.8 Hz, 1H, 1), 7.39 (d, *J* = 7.9 Hz, 1H, 21), 7.20 (t, *J* = 7.7 Hz, 1H, 20), 7.14 (t, *J* = 7.4 Hz, 1H, 2), 6.91 (d, *J* = 7.6 Hz, 1H, 19). LC-MS: *m/z* = 315 [M + H]^+^. Anal. Calcd. for C_14_H_11_ClN_6_O: C, 53.43; H, 3.52; N, 26.70; O, 5.08. Found: C, 53.46; H, 3.49; N, 26.72; O, 5.05.

#### 1-(2-Methyl-6-(1H-tetrazol-5-yl)phenyl)-3-(2-(trifluoromethyl)phenyl)urea (**2.20**)

White crystals, yield: 98%; mp: 255–257°C; IR (cm^−1^): 3316, 1652, 1610, 1591, 1540, 1486, 1465, 1456, 1410, 1320, 1287, 1265, 1232, 1201, 1172, 1162, 1109, 1059, 1046, 1036, 991, 959, 906, 862, 795, 768, 760, 738, 712, 675, 649, 625. ^1^H-NMR: δ (ppm): 16.37 (br.s, 1H, 8), 8.92 (s, 1H, 9), 8.20 (s, 1H, 14), 7.74 (d, *J* = 7.6 Hz, 1H, 3), 7.54 (d, *J* = 8.6 Hz, 1H, 1), 7.48 (m, 2H, 18, 20), 7.40 (d, *J* = 6.6 Hz, 1H, 21), 7.28 (t, *J* = 7.1 Hz, 1H, 19), 7.13 (t, *J* = 6.2 Hz, 1H, 2), 2.35 (s, 3H, CH_3_). LC-MS: *m/z* = 363 [M + H]^+^. Anal. Calcd. for C_16_H_13_F_3_N_6_O: C, 53.04; H, 3.62; N, 23.20; O, 4.42. Found: C, 53.01; H, 3.67; N, 23.16; O, 4.36.

#### 1-(2-Methyl-6-(1H-tetrazol-5-yl)phenyl)-3-(3-(trifluoromethyl)phenyl)urea (**2.21**)

White crystals, yield: 83%; mp: 248–250°C; IR (cm^−1^): 3369, 3236, 3022, 2974, 2892, 2829, 2749, 2639, 2569, 2491, 1660, 1613, 1568, 1555, 1530, 1494, 1480, 1468, 1432, 1331, 1314, 1298, 1264, 1250, 1185, 1170, 1126, 1105, 1073, 1062, 1037, 1000, 920, 891, 857, 785, 754, 725, 695, 677, 652. ^1^H-NMR: δ (ppm): 16.53 (br.s, 1H, 8), 9.50 (s, 1H, 9), 8.62 (s, 1H, 14), 7.86 (s, 1H, 17), 7.64 (d, *J* = 7.4 Hz, 1H, 3), 7.54 (d, *J* = 7.8 Hz, 1H, 1), 7.39 (t, 1H, *J* = 7.8 Hz, 2), 7.35 (d, *J* = 7.8 Hz, 1H, 21), 7.28 (t, *J* = 7.5 Hz, 1H, 20), 7.13 (d, *J* = 7.5 Hz, 1H, 19), 2.35 (s, 3H, CH_3_). LC-MS: *m/z* = 363 [M + H]^+^. Anal. Calcd. for C_16_H_13_F_3_N_6_O: C, 53.04; H, 3.62; N, 23.20; O, 4.42. Found: C, 53.14; H, 3.54; N, 23.29; O, 4.42.

#### 1-(2-Methyl-6-(1H-tetrazol-5-yl)phenyl)-3-(4-(trifluoromethyl)phenyl)urea (**2.22**)

White crystals, yield: 98%; mp: 244–246°C; IR (cm^−1^): 3402, 2928, 1913, 1738, 1694, 1666, 1650, 1603, 1550, 1519, 1502, 1480, 1462, 1444, 1412, 1322, 1232, 1165, 1153, 1111, 1067, 1015, 1000, 985, 949, 911, 843, 822, 785, 773, 748, 729, 720, 676, 624. ^1^H-NMR: δ (ppm): δ 16.51 (br.s, 1H, 8), 9.53 (s, 1H, 9), 8.95 (s, 1H, 14), 8.65 (s, 1H), 7.71 (m, 3H, 3, 17, 21), 7.50 (m, 3H, 1, 18, 20), 7.28 (t, *J* = 7.7 Hz, 1H, 2), 2.35 (s, 2H, CH_3_). LC-MS: *m/z* = 363 [M + H]^+^. Anal. Calcd. for C_16_H_13_F_3_N_6_O: C, 53.04; H, 3.62; N, 23.20; O, 4.42. Found: C, 53.01; H, 3.66; N, 23.15; O, 4.47.

#### 1-(5-Fluoro-2-(1H-tetrazol-5-yl)phenyl)-3-(2-(trifluoromethyl)phenyl)urea (**2.23**)

White crystals, yield: 83%; mp: 248–250°C; IR (cm^−1^): 3301, 3128, 3074, 2992, 2927, 2783, 1674, 1650, 1611, 1592, 1553, 1492, 1454, 1402, 1367, 1319, 1286, 1261, 1247, 1232, 1174, 1127, 1108, 1071, 1059, 1036, 993, 954, 882, 856, 824, 781, 763, 752, 714, 696, 658, 619, 608. ^1^H-NMR: δ (ppm): 16.70 (br.s, 1H, 8), 9.97 (s, 1H, 9), 9.07 (s, 1H, 14), 8.13 (d, *J* = 11.9 Hz, 1H, 6), 7.84 (t, *J* = 7.4 Hz, 1H, 3), 7.66 (m, 3H, 2, 21, 18), 7.32 (t, *J* = 7.3 Hz, 1H, 20), 6.89 (t, *J* = 6.9 Hz, 1H, 19). LC-MS: *m/z* = 367 [M + H]^+^. Anal. Calcd. for C_15_H_10_F_4_N_6_O: C, 49.19; H, 2.75; N, 22.94; O, 4.37. Found: C, 49.24; H, 2.63; N, 22.82; O, 4.32.

#### 1-(5-Fluoro-2-(1H-tetrazol-5-yl)phenyl)-3-(3-(trifluoromethyl)phenyl)urea (**2.24**)

Beige crystals, yield: 51%; mp: 236–238°C; IR (cm^−1^): 3260, 1657, 1650, 1597, 1548, 1491, 1447, 1409, 1337, 1289, 1275, 1223, 1171, 1159, 1111, 1094, 1069, 1023, 990, 930, 897, 872, 820, 808, 797, 776, 755, 742, 694, 669, 634, 618. ^1^H-NMR: δ (ppm): 10.27 (s, 1H, 9), 10.11 (s, 1H, 14), 8.30 (d, *J* = 11.1 Hz, 1H, 6), 7.94 (m, 2H, 3, 17), 7.76 (d, *J* = 7.9 Hz, 1H, 21), 7.41 (t, *J* = 7.8 Hz, 1H, 2), 7.21 (d, *J* = 7.4 Hz, 1H, 20), 6.90 (t, *J* = 7.0 Hz, 1H, 19). LC-MS: *m/z* = 367 [M + H]^+^. Anal. Calcd. for C_15_H_10_F_4_N_6_O: C, 49.19; H, 2.75; N, 22.94; O, 4.37. Found: C, 49.22; H, 2.71; N, 22.96; O, 4.35.

#### 1-(5-Fluoro-2-(1H-tetrazol-5-yl)phenyl)-3-(4-(trifluoromethyl)phenyl)urea (**2.25**)

Beige crystals, yield: 71%; mp: 254–256°C; IR (cm^−1^): 3320, 2920, 2851, 1741, 1694, 1650, 1600, 1538, 1447, 1412, 1321, 1284, 1242, 1225, 1163, 1110, 1067, 1016, 987, 948, 877, 863, 841, 822, 793, 773, 752, 699, 668, 656, 624, 614. ^1^H-NMR: δ (ppm): 10.25 (s, 1H, 9), 10.14 (s, 1H, 14), 8.95 (s, 1H, 6), 7.92 (t, *J* = 7.5 Hz, 1H, 3), 7.71 (m, 2H, 17, 21), 7.6 (m, 1H, 2), 7.51 (m, 2H, 18, 20). LC-MS: *m/z* = 366 [M + H]^+^. Anal. Calcd. for C_15_H_10_F_4_N_6_O: C, 49.19; H, 2.75; N, 22.94; O, 4.37. Found: C, 49.31; H, 2.67; N, 22.91; O, 4.39.

#### 1-(4-Chloro-2-(1H-tetrazol-5-yl)phenyl)-3-(2-(trifluoromethyl)phenyl)urea (**2.26**)

White crystals, yield: 96%; mp: 272–274°C; IR (cm^−1^): 3359, 3296, 1660, 1617, 1595, 1552, 1521, 1507, 1489, 1462, 1448, 1381, 1323, 1306, 1297, 1273, 1245, 1171, 1159, 1141, 1108, 1098, 1081, 1060, 1047, 1036, 1000, 955, 911, 889, 836, 806, 763, 744, 724, 708, 667, 651, 610. ^1^H-NMR: δ (ppm): 16.82 (br.s, 1H, 8), 9.74 (s, 1H, 9), 8.93 (s, 1H, 14), 8.22 (d, *J* = 9.0 Hz, 1H, 6), 7.86 (s, 1H, 3), 7.72 (d, *J* = 7.9 Hz, 1H, 18), 7.62 (d, *J* = 7.6 Hz, 1H, 21), 7.57 (t, *J* = 7.5 Hz, 1H, 20), 7.42 (d, *J* = 8.6 Hz, 1H, 1), 7.28 (t, *J* = 7.2 Hz, 1H, 19). LC-MS: *m/z* = 383 [M + H]^+^. Anal. Calcd. for C_15_H_10_ClF_3_N_6_O: C, 47.07; H, 2.63; N, 21.96; O, 4.18. Found: C, 47.09; H, 2.59; N, 21.93; O, 4.21.

#### 1-(4-Chloro-2-(1H-tetrazol-5-yl)phenyl)-3-(3-(trifluoromethyl)phenyl)urea (**2.27**)

Beige crystals, yield: 53%; mp: 234–238°C; IR (cm^−1^): 3253, 2990, 2926, 2871, 2800, 2780, 2734, 1665, 1606, 1589, 1556, 1532, 1484, 1445, 1393, 1337, 1300, 1287, 1269, 1256, 1241, 1225, 1172, 1158, 1114, 1103, 1071, 1016, 1000, 936, 906, 890, 857, 834, 799, 789, 750, 733, 707, 695, 668, 655. ^1^H-NMR: δ (ppm): 16.89 (br.s, 1H, 8), 10.05 (s, 1H, 9), 10.02 (s, 1H, 14), 8.42 (d, *J* = 8.8 Hz, 1H, 6), 7.96 (s, 2H, 17, 3), 7.73 (d, *J* = 7.3 Hz, 1H, 1), 7.42 (m, 2H, 20, 21), 7.19 (d, *J* = 6.7 Hz, 1H, 19). LC-MS: *m/z* = 383 [M + H]^+^. Anal. Calcd. for C_15_H_10_ClF_3_N_6_O: C, 47.07; H, 2.63; N, 21.96; O, 4.18. Found: C, 47.21; H, 2.54; N, 21.88; O, 4.29.

#### 1-(4-Chloro-2-(1H-tetrazol-5-yl)phenyl)-3-(4-(trifluoromethyl)phenyl)urea (**2.28**)

White crystals, yield: 77%; mp: 255–257°C; IR (cm^−1^): 3118, 3083, 3060, 2924, 2852, 1706, 1679, 1663, 1617, 1605, 1591, 1541, 1510, 1493, 1463, 1450, 1415, 1390, 1321, 1305, 1283, 1267, 1246, 1230, 1219, 1172, 1141, 1122, 1111, 1066, 1042, 1017, 953, 890, 879, 842, 819, 800, 774, 760, 728, 715, 625. ^1^H-NMR: δ (ppm): 16.83 (br.s, 1H, 8), 10.08 (s, 1H, 9), 9.98 (s, 1H, 14), 8.38 (d, *J* = 9.1 Hz, 1H, 6), 7.96 (s, 1H, 3), 7.71 (d, *J* = 7.9 Hz, 2H, 17, 21), 7.50 (d, *J* = 8.3 Hz, 2H, 18, 20), 7.44 (d, *J* = 8.0 Hz, 1H, 1). LC-MS: *m/z* = 383 [M + H]^+^. Anal. Calcd. for C_15_H_10_ClF_3_N_6_O: C, 47.07; H, 2.63; N, 21.96; O, 4.18. Found: C, 47.19; H, 2.59; N, 21.89; O, 4.11.

#### 1-(4-Bromo-2-(1H-tetrazol-5-yl)phenyl)-3-(2-(trifluoromethyl)phenyl)urea (**2.29**)

White crystals, yield: 97%; mp: 270–272°C; IR (cm^−1^): 3355, 3295, 1762, 1663, 1615, 1586, 1551, 1523, 1506, 1458, 1377, 1323, 1306, 1297, 1275, 1242, 1172, 1159, 1141, 1104, 1087, 1059, 1046, 1036, 954, 910, 891, 832, 805, 764, 736, 717, 667, 648, 613. ^1^H-NMR: δ (ppm): 16.78 (br.s, 1H, 8), 9.74 (s, 1H, 9), 8.94 (s, 1H, 14), 8.17 (d, *J* = 8.9 Hz, 1H, 6), 7.99 (s, 1H, 3), 7.71 (d, *J* = 7.8 Hz, 1H, 1), 7.62 (d, *J* = 7.6 Hz, 1H, 21), 7.56 (m, 2H, 18, 20), 7.29 (t, *J* = 7.3 Hz, 1H, 19). Anal. Calcd. for C_15_H_10_BrF_3_N_6_O: C, 42.17; H, 2.36; N, 19.67; O, 3.75. Found: C, 42.14; H, 2.39; N, 19.63; O, 3.79.

#### 1-(4-Bromo-2-(1H-tetrazol-5-yl)phenyl)-3-(3-(trifluoromethyl)phenyl)urea (**2.30**)

Beige crystals, yield: 61%; mp: 269–271°C; IR (cm^−1^): 3289, 3009, 2984, 2924, 2867, 2798, 2731, 1664, 1606, 1583, 1556, 1527, 1484, 1446, 1391, 1337, 1301, 1286, 1269, 1241, 1172, 1156, 1112, 1073, 1012, 1001, 937, 890, 867, 831, 799, 786, 749, 723, 695, 667, 635. ^1^H-NMR: δ (ppm): 16.88 (br.s, 8), 10.05 (s, 1H, 9), 10.03 (s, 1H, 14), 8.37 (d, *J* = 9.1 Hz, 1H, 6), 8.10 (s, 1H, 3), 7.96 (s, 1H, 17), 7.73 (d, *J* = 7.9 Hz, 1H, 1), 7.56 (d, *J* = 8.7 Hz, 1H, 21), 7.40 (t, *J* = 7.6 Hz, 1H, 20), 7.20 (d, *J* = 7.3 Hz, 1H, 19). LC-MS: *m/z* = 429 [M + H]^+^. Anal. Calcd. for C_15_H_10_BrF_3_N_6_O: C, 42.17; H, 2.36; N, 19.67; O, 3.75. Found: C, 42.19; H, 2.29; N, 19.69; O, 3.89.

#### 1-(4-Bromo-2-(1H-tetrazol-5-yl)phenyl)-3-(4-(trifluoromethyl)phenyl)urea (**2.31**)

White crystals, yield: 69%; mp: 256–258°C; IR (cm^−1^): 3295, 3260, 3188, 3131, 3073, 1724, 1678, 1604, 1584, 1554, 1516, 1487, 1410, 1379, 1327, 1301, 1281, 1267, 1235, 1186, 1165, 1113, 1067, 1017, 1010, 951, 898, 878, 853, 837, 805, 795, 758, 740, 710, 693, 623, 611. ^1^H-NMR: δ (ppm): 16.73 (br.s, 1H, 8), 10.09 (s, 1H, 9), 10.00 (s, 1H, 14), 8.33 (d, *J* = 9.0 Hz, 1H, 6), 8.10 (s, 1H, 3), 7.68 (m, 3H, 1, 17, 21), 7.57 (m, 2H, 18, 20). LC-MS: *m/z* = 427 [M + H]^+^. Anal. Calcd. for C_15_H_10_BrF_3_N_6_O: C, 42.17; H, 2.36; N, 19.67; O, 3.75. Found: C, 42.21; H, 2.32; N, 19.72; O, 3.71.

### General Procedure for the Synthesis of R^1^-tetrazolo[1,5-c]quinazolin-5(6H)-ones (3.1–3.5)

One g (0.0062 mol) of 2-(1*H*-Tetrazol-5-yl)aniline was dissolved in acetic acid after 0.74 g (0.0062 mol) of isocyanatobenzene was added (any other substitute of isocyanatobenzene gave the same product of cyclization). The mixture was refluxed for 2 h and cooled down. The formed precipitate was filtered, washed with water, dried, and crystallized from a mixture of propane-2-ol:water (1:1).

#### Tetrazolo[1,5-c]quinazolin-5(6H)-one (**3**.**1**)

Beige crystals, yield: 96%; mp: 295–297°C; IR (cm^−1^): 3147, 3114, 3085, 3047, 2974, 2916, 2849, 2755, 2709, 1746, 1714, 1660, 1625, 1587, 1547, 1515, 1483, 1463, 1442, 1430, 1415, 1388, 1342, 1304, 1256, 1202, 1167, 1157, 1114, 1088, 1025, 995, 969, 896, 880, 810, 783, 757, 742, 731, 709, 698, 675, 658, 623. ^1^H-NMR: δ (ppm): 12.68 (s, 1H, NH), 8.27 (d, *J* = 7.8 Hz, 1H, H-10), 7.71 (t, *J* = 7.7 Hz, 1H, H-9), 7.50 (d, *J* = 8.2 Hz, 1H, H-7), 7.42 (t, *J* = 7.5 Hz, 1H, H-8). LC-MS: *m/z* = 187 [M + H]^+^. Anal. Calcd. for C_8_H_5_N_5_O: C, 51.34; H, 2.69; N, 37.42; O, 8.55. Found: C, 51.43; H, 2.54; N, 37.57; O, 8.46.

#### 7-Methyltetrazolo[1,5-c]quinazolin-5(6H)-one (**3.2**)

White crystals, yield: 21%; mp: 265–267°C; IR (cm^−1^): 3302, 3209, 3153, 3049, 2955, 2916, 2848, 1743, 1723, 1635, 1594, 1565, 1531, 1498, 1479, 1426, 1367, 1337, 1249, 1219, 1182, 1105, 1069, 1055, 1032, 999, 956, 910, 869, 843, 799, 779, 768, 745, 687, 629. ^1^H-NMR: δ (ppm): 10.11 (s, 1H, NH), 8.12 (d, *J* = 7.9 Hz, 1H, H-10), 7.33 (m, 2H, H-8, 9), 2.36 (s, CH_3_). Anal. Calcd. for C_9_H_7_N_5_O: C, 53.73; H, 3.51; N, 34.81; O, 7.95. Found: C, 53.79; H, 3.37; N, 34.89; O, 7.85.

#### 8-Fluorotetrazolo[1,5-c]quinazolin-5(6H)-one (**3.3**)

Beige crystals, yield: 50%; mp: 254–256°C; IR (cm^−1^): 3531, 3395, 3144, 3086, 2926, 1730, 1674, 1630, 1596, 1539, 1509, 1470, 1444, 1350, 1289, 1254, 1202, 1162, 1142, 1098, 1015, 967, 859, 816, 749, 697, 666, 643. ^1^H-NMR: δ (ppm): 12.79 (s, 1H, NH), 8.32 (dd, *J* = 9.3, 5.6 Hz, 1H, H-10), 7.20 (m, 2H, H-7,9). Anal. Calcd. for C_8_H_4_FN_5_O: C, 46.84; H, 1.97; N, 34.14; O, 7.80. Found: C, 46.89; H, 1.87; N, 34.01; O, 7.89.

#### 9-Chlorotetrazolo[1,5-c]quinazolin-5(6H)-one (**3.4**)

Beige crystals, yield: 74%; mp: 270–272°C; IR (cm^−1^): 3242, 3184, 2958, 2916, 2850, 1762, 1724, 1683, 1654, 1645, 1628, 1591, 1549, 1498, 1466, 1444, 1407, 1374, 1341, 1278, 1242, 1203, 1161, 1131, 1096, 1081, 1040, 1026, 969, 927, 905, 886, 830, 776, 762, 736, 696, 662. ^1^H-NMR: δ (ppm): 12.78 (s, 1H, NH), 8.22 (s, 1H, H-10), 7.68 (d, *J* = 7.5 Hz, 1H, H-8), 7.49 (d, *J* = 8.8 Hz, 1H, H-7). Anal. Calcd. for C_8_H_4_ClN_5_O: C, 43.36; H, 1.82; N, 31.60; O, 7.22. Found: C, 43.43; H, 1.77; N, 31.63; O, 7.18.

#### 9-Bromotetrazolo[1,5-c]quinazolin-5(6H)-one (**3.5**)

Beige crystals, yield: 80%; mp: 272–274°C; IR (cm^−1^): 3190, 3100, 3068, 3030, 2955, 2917, 2849, 1715, 1697, 1651, 1624, 1588, 1547, 1502, 1463, 1435, 1407, 1371, 1340, 1277, 1238, 1201, 1130, 1101, 1070, 1023, 970, 902, 888, 828, 762, 735, 692, 662. ^1^H-NMR: δ (ppm): 12.80 (s, 1H, NH), 8.36 (s, 1H, H-10), 7.81 (d, *J* = 7.0 Hz, 1H, H-8), 7.44 (d, *J* = 8.0 Hz, 1H, H-7). Anal. Calcd. for C_8_H_4_BrN_5_O: C, 36.12; H, 1.52; N, 26.32; O, 6.01. Found: C, 36.26; H, 1.39; N, 26.46; O, 5.88.

### Pharmacology

White Wistar rats weighing 260–280 g of 3.5 months age were used to accomplish the experimental studies. They were obtained from the nursery «Biomodelservis». The animals were kept on a standard diet with the natural change of day and night and free access to water and food. All experimental procedures were carried out according to the «Regulations on the Use of Animals in Biomedical Research».

After the quarantine, pre-individually marked animals were randomly divided into groups of six male rats, provided with the absence of external signs of disease and homogeneity groups by weight (±15%).

#### Preliminary Screening

Rats were starved overnight before oral administration of the test substances. Each laboratory rat was weighed before the experiment was determined. Intragastric administration of the substances was done in water solution or a finely dispersed suspension stabilized by Tween 80 in the dose of 50 mg/kg. Intact and control groups obtained equivalent volumes of water by the same way. Hypoglycemic action of the synthesized compounds was evaluated via changes in glucose level before and after administration of the studied compounds. Measurements of the glucose levels of six rats for each compound were carried out in 2, 4, 6, and 8 h after administration [17].

The concentration change in blood glucose after a single oral administration was used in order to evaluate the possible hypoglycemic activity of the new substances. Determination of blood glucose was performed using a blood glucose meter «One Touch Select». For evaluation of hypoglycemic activity, the dynamic area under the curve was calculated. Z coordinated time (2, 4, 6, 8 h) and y coordinated the percentage of glucose level decrease.

Primary insulin resistance was induced by daily intramuscular injection of a glucocorticoid, namely Dexamethasone in the dose of 0.125 mg/kg during 13 days [18, 19]. The state of glucose homeostasis was evaluated by the values of basal glycemia and carbohydrate tolerance, which were defined via the oral test for glucose tolerance, rapid insulin, and adrenaline tests [18, 19]. «Metformin» (50 and 200 mg/kg) and «Gliclazide» (50 mg/kg) were used as reference drugs.

#### Oral Glucose Tolerance Test

Glucose (2 g/kg) was intragastrically administrated via a noninvasive probe. Blood samples for glucose analysis were collected before administration and after 15, 30, 60, and 120 min.

#### Rapid Insulin Test

Insulin was intraperitoneally injected into animals (1 unit/kg). Blood samples for glucose analysis were collected before administration and after 30 min.

#### Adrenalin Test

0.18% adrenaline solution at a dose of 0.5 mg/kg was administered to rats. Blood samples for glucose analysis were collected before administration, then after 30 and 90 min.

For the oral glucose tolerance and adrenaline tests, the area under the curve was calculated as following: z coordinated min, y coordinated glucose levels.

Statistical data processing was performed using a standard statistical package program, Version «MicrosoftOfficeExcel 2003», «STATISTICA® forWindows 6.0» (StatSoftInc., No AXXR712D833214FAN5). Arithmetic mean (M) and standard error of the mean (±m) were calculated for each of the studied parameters. During verification of the statistical hypothesis, the null hypothesis was declined if the statistical criterion was p<0.05.

### Docking

Research was conducted by flexible molecular docking as an approach to finding molecules with affinity to a specific biological target.

#### Ligand Preparation

Substances were drawn using MarvinSketch 6.3.0 and saved in mol format [20]. Afterwards, they were optimized by the program HyperChem8.0.8 using a molecular mechanical MM+ algorithm combined with semi-empirical PM3 molecular modeling method with a maximum number of cycles and Polak-Ribiere (Conjugate Gradient) algorithm. Molecular mechanics has been used to produce more realistic geometry values for the majority of organic molecules owing to the fact of being highly parameterized. The next step was a re-optimization of the MM+ optimized structures by applying a semi-empirical PM3 molecular modeling method and saved as pdb-files. Using AutoDockTools-1.5.6, pdb-files were converted to PDBQT and the number of active torsions was set as default [21].

#### Protein Preparation

PDB files were downloaded from the protein data bank. Discovery Studio 4.0 was used to delete water molecules and ligand from crystal. Proteins were saved as pdb-files. In AutoDockTools-1.5.6, polar hydrogens were added and saved as PDBQT. The grid box was set as following: center_x = -23.247, center_y = 41.137, center_z = 5.939, size_x = 12, size_y = 14, size_z = 10 for 3QQP; center_x = 16.251, center_y = 6.145, center_z = 45.867, size_x = 14, size_y = 18, size_z = 20 for 2XKW; center_x = 51.123, center_y = 48.163, center_z = 37.781, size_x = 16, size_y = 12, size_z = 12 for 2RGU. Vina was used to carry docking [22]. For visualization, Discovery Studio 4.0 was used.
